# Technical note: A comparison of physician doses in C‐Arm and CT fluoroscopy procedures

**DOI:** 10.1002/acm2.14335

**Published:** 2024-03-27

**Authors:** Jonathan Troville, Emily Knott, Carlos Alberto Reynoso‐Mejia, Martin Wagner, Fred T. Lee, Timothy P. Szczykutowicz

**Affiliations:** ^1^ Departments of Medical Physics University of Wisconsin‐Madison Madison Wisconsin USA; ^2^ Departments of Radiology University of Wisconsin‐Madison Madison Wisconsin USA; ^3^ Departments of Cleveland Clinic Medical School University of Wisconsin‐Madison Madison Wisconsin USA

**Keywords:** computed tomography, fluoroscopy, physician dose, radiation safety

## Abstract

**Purpose:**

We address the misconception that the typical physician dose is higher for CT fluoroscopy (CTF) procedures compared to C‐Arm procedures.

**Methods:**

We compare physician scatter doses using two methods: a literature review of reported doses and a model based on a modified form of the dose area product (DAP). We define this modified form of DAP, “cumulative absorbed DAP,” as the product of the area of the x‐ray beam striking the patient, the dose rate per unit area, and the exposure time.

**Results:**

The patient entrance dose rate for C‐Arm fluoroscopy (0.2 mGy/s) was found to be 15 times lower than for CT fluoroscopy (3 mGy/s). A typical beam entrance area for C‐Arm fluoroscopy reported in the literature was found to be 10.6 × 10.6 cm (112 cm^2^), whereas for CTF was 0.75 × 32 cm (24 cm^2^). The absorbed DAP rate for C‐Arm fluoroscopy (22 mGy*cm^2^/s) was found to be 3.3 times lower than for CTF (72 mGy*cm^2^/s). The mean fluoroscopy time for C‐Arm procedures (710 s) was found to be 21 times higher than for CT fluoroscopy procedures (23 s). The cumulative absorbed DAP for C‐Arm procedures was found to be 9.4 times higher when compared to CT procedures (1.59 mGy*m^2^ vs. 0.17 mGy*m^2^).

**Conclusions:**

The higher fluoroscopy time in C‐Arm procedures leads to a much lower cumulative DAP (i.e., physician scatter dose) in CTF procedures. This result can inform interventional physicians deciding on whether to perform inter‐procedural imaging inside the room as opposed to retreating from the room.

## 1 INTRODUCTION

Physician dose in interventional CT fluoroscopy (CTF) procedures is an ongoing concern due to non‐optimal use of protective attire,^1^ inconsistent use of dosimeters,^2^ and misconceptions about how doses compare to those in C‐Arm fluoroscopy procedures among interventional radiologists.^3^, ^4^, ^5^ CT vendors include options for the acquisition of real‐time, continuous fluoroscopic images as well as sequential images by performing intermittent “taps” of the fluoroscopic pedal. However, while these options are available, concerns with scatter dose due to the relatively high dose rates in these procedures prevail. Therefore, it is common for intra‐procedural imaging to be performed with the physician outside of the room.^6^ Performing intra‐procedural imaging while retreated from the room (1) forces the procedural team to move in and out of the room, which means exam times are longer and (2) increases patient radiation dose as most sites use non‐interventional imaging modes (i.e., helical/spiral imaging as opposed to small collimation axial scanning) when the physician is retreated from the room.^7^, ^8^

A paucity of literature exists comparing physician scatter dose between interventional procedures performed using c‐arm scanners and MDCT. Increasing concern over physician dose in interventional settings is warranted given the potential association of cataractogenesis and oncogenesis among physicians performing interventional procedures.^9^, ^10^ As such, the most encountered method for performing interventional CTF procedures is for all the staff to leave the room during a scan; which differs from typical C‐Arm‐based interventions where the physician remains in the room.^6^

The work presented in this study, to our knowledge, is the first of its kind; an analysis which ties in commonly encountered dose metrics as reported in the literature and how they contribute to total scatter production for the physician.

## 2 MATERIALS AND METHODS

Our study addresses physician scatter dose in two ways: (1) we used data collected from a previous literature review on physician scatter dose between CTF and C‐Arm interventional procedures and (2) we developed a new physician scatter dose surrogate (i.e., cumulative absorbed DAP) to predict the ratio of C‐Arm to CTF physician scatter doses. For cumulative absorbed DAP, we also performed a small literature search and pulled data from our own institution to provide technique statistics to feed into the model.

### 2.1 Literature review on physician doses in C‐Arm and CTF procedures

We used a Society of Interventional Radiology literature review to obtain typical physician scatter doses.^11^ Briefly, Knott et al. (2022) analyzed 15 studies acquired from the following search words: aortic repair, urethrogram, ERCP, antegrade, HSG, barium swallow, cystogram, transhepatic biliary drainage and stents, and percutaneous vertebroplasty procedures.^11^ This literature review pulled personal dose equivalents or entrance air kerma values for C‐Arm and CTF procedures, which provided the basis of comparison to our study for relative scatter production.

### 2.2 Estimation of absorbed DAP rates in C‐Arm and CTF procedures

Information from abdominothoracic or phantom‐equivalent scans was extracted from three papers to deduce common absorbed dose area product (DAP) rates.^12^, ^13^, ^14^ Key terms in the search were: entrance beam size, C‐Arm fluoroscopy, staff dose, and interventional CTF.

An estimate of typical patient skin entrance dose rates for C‐Arm procedures was acquired from a paper by Zweers et al. (1998).^12^ In this paper, patient entrance dose rates were compared across hospitals for TIPS procedures. Since TIPS procedures are not representative of most C‐Arm procedures, we extracted an average skin entrance field size for cardiac procedures from a paper by Vano et al. (2001).^13^ We acquired dose rate information for CTF procedures from Teeuwisse et al. (2001).^14^ Teeuwisse et al. (2001) includes dose estimates for a 32 cm body phantom, which yields a reasonable one‐to‐one comparison with the dose rates encountered in abdominal or cardiac procedures.^14^

Hospital A from the Zweers et al. (1998) study represented the control group for which only automatic brightness control (ABC) was used to adjust technique parameters.^12^ Therefore, we utilized data from this cohort since it is most representative of the typical practice in C‐Arm procedures when compared to the alternatives: (1) manual adjustment of parameters and (2) a combination of ABC and manual parameter adjustment. We only extracted data related to fluoroscopic projections resulting in a median entrance skin dose rate of 50 mGy/min (0.83 mGy/s). This dose rate was reported for an entrance field area of 21 × 21 cm (441 cm^2^), which is representative of TIPS procedures. A field size of 10.6 × 10.6 cm (approximately 112 cm^2^) for cardiac procedures was extracted from the Vano et al. study.^13^ We used this more common cardiac beam area to normalize the dose rate of Zweers et al. (1998).^12^ Correcting 0.83 mGy/s by the ratio of 112 and 441 cm^2^ yields an entrance dose rate of approximately 0.2 mGy/s.

### 2.3 Institution specific CTF procedure data

We pulled CT cases performed from March of 2016 to March of 2020. Interventional cases were filtered from diagnostic cases using the study description. Included descriptions were: “ablation,” “abscess drain,” “aspiration CT guided,” “biopsy CT guided,” “spine nerve root block,” and “spine sacroiliac injection.” We calculated the averages and 25th/50th/75th percentiles for: number of taps, beam energy, rotation time, and tube current. We calculated this usage data with fields obtained from our commercial dose monitoring system (DoseWatch, GE Healthcare Chicago Illinois).

### 2.4 Cumulative absorbed DAP

To compare scatter radiation between modalities, we define a new metric in this paper. The “cumulative absorbed dose area product (DAP)” is the product of absorbed dose at the surface of a patient and the beam area at the surface of the patient. In comparison, the standard definition of DAP, which is synonymous to kerma area product (KAP), is the product of the air kerma at a given distance from the source and the beam area at the same location. Cumulative absorbed DAP accounts for the main contributors to physician scatter dose: (1) the larger the field value of irradiated patient tissue the higher the physician scatter dose; (2) the longer the period of irradiation, the higher the physician scatter dose; and (3) the higher the dose rate the higher the physician scatter dose.

## 3 RESULTS

**FIGURE 1:**
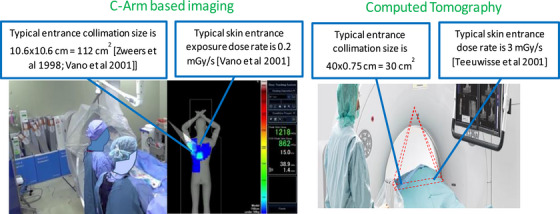
A comparison of typical patient entrance beam sizes and dose rates in C‐Arm and CTF interventional procedures.

Figure 1 summarizes the typical patient entrance beam sizes and dose rates for C‐Arm and CTF procedures extracted from Zweers et al. (1998), Vano et al. (2001), and Teeuwisse et al. (2001).^12^, ^13^, ^14^ This figure also highlights differences in beam geometry, gantry configuration, and in‐room staff involvement observed during these procedures. For real‐time CTF, wide (i.e., in the fan direction of the beam) and narrow (i.e., in the cone direction of the beam) beams are typically employed. CTF beam size is 40 × 0.75 cm (30 cm^2^) as compared to a typical entrance collimation size of 10.6 × 10.6 cm (112 cm^2^) in C‐Arm procedures. Furthermore, our literature review indicates that the entrance dose rates encountered in CTF procedures are much larger than in C‐Arm procedures with mean values of 3 and 0.2 mGy/s, respectively.

Table 1 provides a summary of typical dose rates for C‐Arm and CTF procedures as well as the exposed entrance area on the patient, absorbed DAP rate, mean fluoroscopy time, and cumulative absorbed DAP. The absorbed DAP rate was computed by multiplying the literature‐reported entrance dose rates and the exposure areas. We then calculated the cumulative absorbed DAP for each modality by multiplying each absorbed DAP rate by the corresponding fluoroscopy time. These results indicate the following for CTF compared to C‐Arm procedures: (1) 15 times more patient entrance dose per second, (2) 4.7 times less exposure area, (3) 3.3 times more DAP per second, and (4) 30.9 times less fluoroscopy time. These comparisons yield a total reduction of 9.4 times less cumulative DAP for CTF relative to C‐Arm procedures.

**TABLE 1:** A comparison of physician scatter dose‐related metrics for C‐Arm and CT interventional procedures.  Zweers et al. (1998) was utilized for estimating entrance dose rates in C‐Arm procedures, Vano et al. (2001) was utilized for estimating the exposed area (skin entrance beam area) in C‐Arm procedures, and Teeuwisse et al. (2001) was utilized for estimating entrance dose rates in CTF procedures.^12^, ^13^, ^14^ The mean fluoroscopy time for C‐Arm procedures was extracted from the Knott et al. (2022) literature review, and the mean fluoroscopy time for CTF procedures was extracted from the Knott et al. (2022) analysis of 1722 procedures.^11^ Mean air kerma rate and mean total air kerma were also extracted from the Knott et al. (2022) study.^11^

*Model based Comparison: Predicted Physician Scatter Dose based on “cumulative absorbed DAP”* ^12^, ^13^, ^14^		
	C‐Arm	CT
Entrance Dose Rate (mGy/s)	0.2	3.0
Exposed Area (cm^2^)	112	24
Absorbed DAP Rate (mGy*cm^2^/s)	22	72
Mean Fluoroscopy Time (sec)*	710	23
Cumulative Absorbed DAP (mGy*m2)	1.59	0.17
Ratio of C‐Arm to CT Cumulative Absorbed DAP	9.4	

*Literature Review based Comparison: Predicted Physician Scatter Dose based on “mean total air kerma* ^11^ *”*				
Procedure Type	Mean Fluoroscopy Time (sec)	Mean Air Kerma Rate (uGy/s)	Mean Total Air Kerma (uGy)	Ratio of C‐Arm to CT Mean Total Air Kerma
CTF	22.5 ± 8.3	0.80 ± 0.56	18.0	9.1
C‐Arm	710.4 ± 51.3	0.23 ± 0.31	163.4	

Summary technique statistics from the Knott et al. (2022) literature review of 15 publications on C‐Arm procedures are shown in Table 1 and demonstrate that physician scatter doses are 9.1 times higher for C‐Arm procedures relative to CTF procedures.^11^ Table 1 also summarizes 1722 CTF procedures from our institution. We found: 24 CTF taps per case (inter quartile range (IQR) of 10−40), 22.5 s average tube on time, 120 kV (IQR of 120–120), 5 mm collimation (IQR of 5−10), 60 mA (IQR of 40−60), and 0.5 s rotation time (IQR of 0.5–0.5). Using these parameters and a correction for lead shielding, the physician dose calculated for 10, 24, and 40 taps was 0.20, 0.48, and 0.81 µGy (select comparison studies in Table 1).

## 4 DISCUSSION

Our model prediction using cumulative absorbed DAP matches well with the Knott et al. (2022) literature review. The ratio of our physician dose rate surrogate, cumulative absorbed DAP, was 9.4 which compares well with the Knott et al. (2022) value of 9.1.^11^

In our experience, most physicians believe CTF leads to a larger physician dose compared to C‐Arm interventional procedures. Our results indicate CTF leads to a much larger entrance dose rate when compared to C‐Arm procedures (3.0 mGy/s vs. 0.2 mGy/s), as well as a significantly larger absorbed DAP rate (72 mGy*cm^2^/s vs. 22 mGy*cm^2^/s). However, the cumulative DAP (i.e., surrogate for physician scatter dose) is much lower (0.17 mGy*m^2^ vs. 1.59 mGy*m^2^) for CTF procedures comparatively. It is clear from our analysis that the difference in fluoroscopy time (22.5 s vs. 710.4 s) for these procedures contributes to much higher physician scatter doses in C‐Arm‐based procedures (compared with CTF).

The comparison of the cumulative absorbed DAP ratio in this study to the ratio of cumulative physician doses in other studies depends strongly on the validity of reported typical physician dose rates for CTF and C‐Arm procedures. Various studies have been performed to analyze typical physician doses in both C‐Arm and CTF procedures. For C‐Arm procedures, physician dose rates at the collar level may be in the range of 0.20–0.83 mGy/h depending on the entrance beam area, patient size, and patient position relative to the beam and physician.^15^ Similarly, for CTF procedures, dose rates at a typical physician's chest location have been found to be in the range of 3.6–59.4 mGy/h depending on collimation size and physician location with respect to the tube.^4^ The mean physician dose rate of 2.88 ± 2.02 mGy/h reported by the Knott et al. literature review in 2022 for CTF procedures, which is contained in Table 1 of this study, compares well to the measurements by Knott et al. in 2020 depending on how partial angle scanning is considered.^2^, ^11^ Finally, the literature‐reported range of physician dose rates in C‐Arm procedures in the Knott et al. (2022) study compares well to those reported in the Schueler et al. study in 2006.^11^, ^15^

One limitation of our study is the geometry for where the physician stands during a C‐arm versus CTF procedure. The distances between the patient and physician vary from C‐arm to CTF because of the physical sizes and patient access limitations of the respective scanners. In C‐Arm procedures, the interventionalist could be approximately 74 cm from the scatter source, while in CTF procedures this distance is closer to 85 cm when considering the bulkiness of the CT gantry. The Knott et al. (2022) literature review inherently accounts for this difference since all recorded dose rates and calculations were performed with these differences in scatter source (i.e., the patient) to physician distance.^11^

In the current study, geometric differences are accounted for in the cumulative DAP calculations through considerations for both the exposed area and entrance dose rate estimates. Patient entrance dose rates depend strongly on the source‐to‐skin distance, which is accounted for inherently in both C‐arm and CTF estimates. Source‐to‐skin distance in both types of procedures depends on patient size and patient bed position. Since the entrance dose rate in CTF procedures reflects an average over the patient surface, we reported values for an average‐sized patient with an effective diameter of 32 cm. C‐Arm procedures can be difficult for obtaining entrance skin dose estimates due to the dynamics of both C‐Arm and table motion. The entrance dose rates extracted from Zweers et al. (1998) capture values averaged over various procedures and thus inherently account for the complex geometric dynamics.^12^ Additionally, our literature review captures procedure‐averaged exposed areas as well as phantom‐study average exposed areas, which yields estimates for typical beam area variations.

## 5 CONCLUSION

This paper highlights a comparison of physician doses in C‐Arm and CT interventional procedures using cumulative absorbed DAP as an estimate of the relative amount of scattered radiation produced. The novel approach discussed in this paper indicates that C‐Arm procedures lead to physician doses which are 9.4 times higher than for CTF procedures, which compares well with a literature‐reported ratio of 9.1. Our hope is for this work to address misconceptions about the relative amount of dose between C‐Arm and CTF modalities. Clinically, better knowledge of physician doses could help keep physicians “bedside” during interventional procedures.

## AUTHOR CONTRIBUTIONS

Jonathan Troville and Timothy P. Szczykutowicz proposed the concepts for performing this work. Jonathan Troville developed the absorbed cumulative DAP metric and performed the literature reviews relevant to calculations of this metric. Emily Knott performed the literature reviews necessary for comparing the work in this manuscript to prior work. Carlos Alberto Reynoso‐Mejia contributed significantly to the understanding, computation, and validation of the methods in this work and the prior work to which we compare. Martin Wagner contributed to the understanding and validity of the methods in this work. Fred T. Lee contributed to the communication and soundness of the results in both technical and clinical contexts. All the authors contributed to the drafting of the manuscript.

## CONFLICT OF INTEREST STATEMENT

The authors declare no conflicts of interest.
